# Microbiome influence in gastric cancer progression and therapeutic strategies

**DOI:** 10.3389/fmed.2025.1681824

**Published:** 2025-10-20

**Authors:** Jhommara Bautista, Ivan Maldonado-Noboa, Daniela Maldonado-Guerrero, Lisette Reinoso-Quinga, Andrés López-Cortés

**Affiliations:** ^1^Cancer Research Group (CRG), Faculty of Medicine, Universidad de Las Américas, Quito, Ecuador; ^2^Jefe de Servicio de Oncología, Hospital Metropolitano de Quito, Quito, Ecuador

**Keywords:** gastric microbiome, gastric cancer, biology, mechanisms, epidemiology, biomarkers, therapeutic modulation, stomach neoplasms

## Abstract

Gastric cancer (GC) remains a major global health burden, ranking as the fifth most commonly diagnosed malignancy and the fourth leading cause of cancer-related death worldwide. While *Helicobacter pylori* is established as the primary microbial risk factor, emerging evidence underscores the broader oncogenic potential of gastric microbiome dysbiosis. This review synthesizes recent advances in understanding how microbial communities, both within the stomach and along the gut–stomach axis, contribute to gastric carcinogenesis. We explore how alterations in microbial diversity, virulence, and metabolic output disrupt mucosal homeostasis, drive chronic inflammation, and reshape local immune surveillance. Special attention is given to the molecular mechanisms by which *H. pylori* virulence factors cytotoxin-associated gene A (CagA) and VacA, vacuolating cytotoxin, induce epithelial transformation, immune evasion, and epigenetic reprogramming. We also highlight the oncogenic roles of non-*H. pylori* taxa such as *Fusobacterium nucleatum*, *Streptococcus anginosus*, and *Lactobacillus fermentum*, which synergize with host and environmental factors to sustain tumor-promoting microenvironments. Multi-omics studies reveal microbial signatures predictive of disease progression, therapeutic response, and prognosis, laying the foundation for microbiome-informed precision oncology. Furthermore, we examine how microbiota-targeted interventions, probiotics, prebiotics, dietary modulation, and fecal microbiota transplantation, can enhance chemotherapy and immunotherapy efficacy while mitigating treatment-related toxicity. Lastly, we discuss the implications of early *H. pylori* eradication, the impact of antibiotic resistance, and the need for global surveillance strategies.

## Introduction

Gastric cancer (GC), refers to malignant neoplasms arising from the epithelial lining of the stomach, excluding tumors located at the esophagogastric junction (1). According to GLOBOCAN 2022, an estimated 970,231 new cases of gastric cancer and 673,007 related deaths were reported worldwide, making it the fifth most diagnosed cancer and the fourth leading cause of cancer death globally, accounting for approximately 7.5% of all cancer deaths. The highest incidence rates were observed in Eastern Asia, particularly in Mongolia (32.5 per 100,000), Japan (26.4 per 100,000), and the Republic of Korea (27.4 per 100,000). Other high-incidence regions include Central and Eastern Europe, South America (notably Chile and Colombia), and parts of Central Asia. In contrast, incidence rates remain substantially lower in North America (3.1 per 100,000), Northern Europe, and most African countries (typically below 5 per 100,000). Mortality patterns closely mirrored incidence trends, with the highest gastric cancer death rates occurring in Mongolia (26.9 per 100,000) and Central Asian countries, while significantly lower rates (<3 per 100,000) were seen in much of Northern Europe, North America, and sub-Saharan Africa (2). The global burden remains substantial due to delayed diagnosis and the limited effectiveness of treatments in advanced metastatic stages (3).

At the molecular level, GC is a highly heterogeneous malignancy characterized by diverse genomic and epigenetic alterations. The Cancer Genome Atlas (TCGA) categorizes GC into four primary molecular subtypes: Epstein–Barr virus-positive, microsatellite instability-high, genomically stable, and chromosomal instability, each exhibiting distinct biological behaviors and clinical outcomes. These classifications inform both therapeutic decisions and prognostic assessments. MSI-H tumors, in particular, show elevated mutational burden and immune infiltration, which translate into enhanced sensitivity to immune checkpoint inhibitors and support their prioritization in immunotherapy-based approaches (4–6).

**Figure 1:**
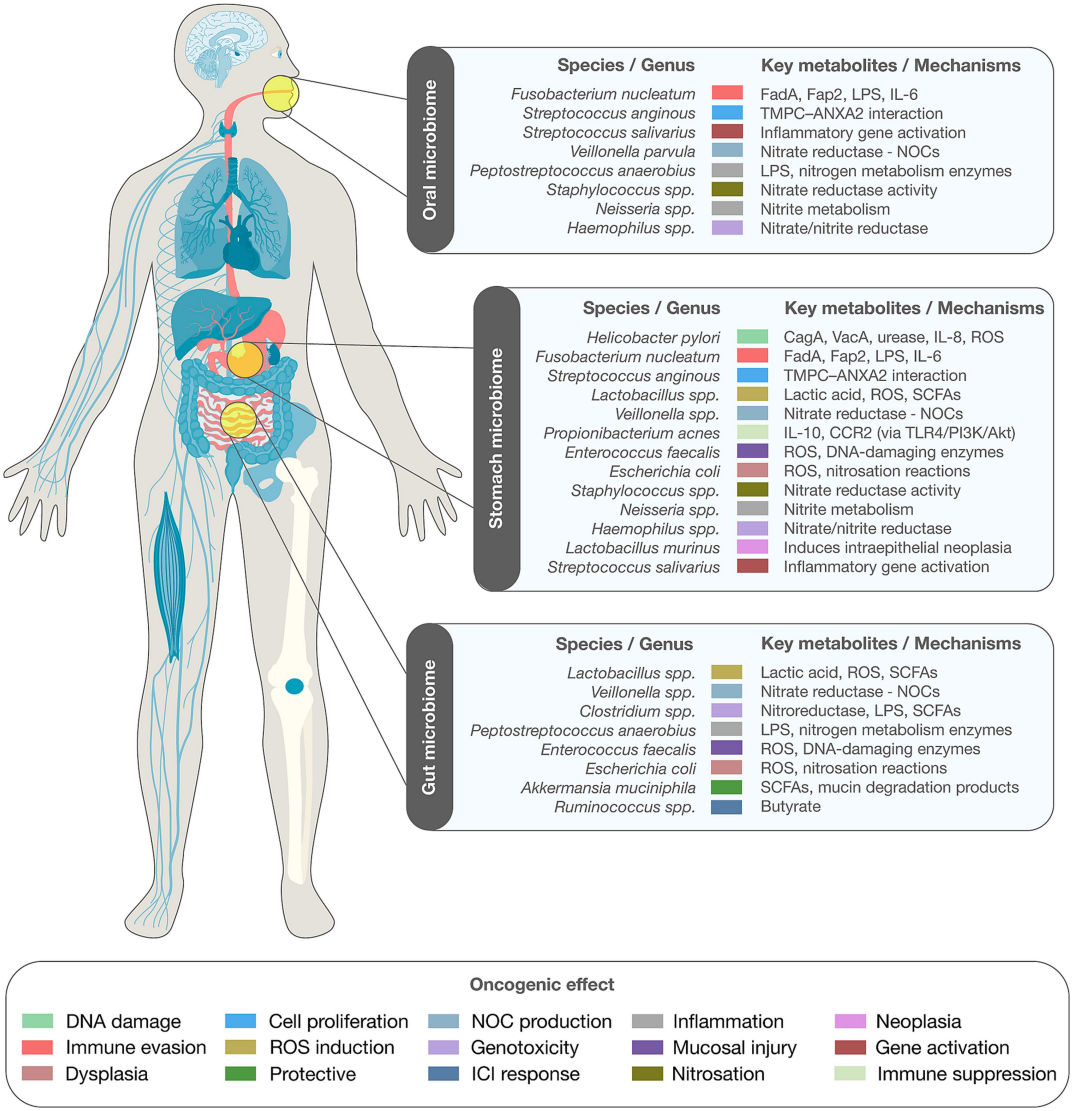
Landscape of the gastric cancer microbiome. The schematic depicts bacterial taxa originating from the oral cavity, stomach, and gut, alongside representative microbial metabolites and molecular pathways implicated in tumorigenesis.

While *H. pylori* remains the predominant etiological agent in gastric carcinogenesis, recent research highlights the broader contribution of gastric microbiome dysbiosis to tumor progression. Alterations in microbial diversity and structure have been implicated in promoting chronic mucosal inflammation, disrupting epithelial integrity, and impairing local immune regulation—factors that together foster a pro-oncogenic gastric microenvironment (7, 8) (Figure 1).

In parallel with its biological complexity, GC poses a major socioeconomic challenge, particularly in low- and middle-income countries where the absence of widespread endoscopic screening often results in delayed diagnosis and poor outcomes (9). Additionally, structural inequities in access to cancer care, driven by geographic disparities, insurance coverage gaps, and limited availability of advanced therapeutics, remain persistent obstacles to global cancer control (10). To ensure comprehensive coverage of these issues, we conducted a targeted narrative search to identify primary and review literature on the gastric microbiome and gastric cancer, focusing on biology, mechanisms, epidemiology, biomarkers, and therapeutic modulation. Databases consulted included PubMed/MEDLINE, Embase, Web of Science, Scopus, the Cochrane Library, and ClinicalTrials.gov for ongoing or registered trials. Google Scholar was additionally used for citation tracking. The main time window was January 2010 to September 2025, although earlier seminal studies were considered when foundational for mechanistic context. Searches were restricted to English. Boolean search strings included combinations of “gastric cancer” OR “stomach neoplasms” with “microbiome” OR “microbiota” OR “dysbiosis” OR “*Helicobacter pylori*” AND “carcinogenesis” OR “immune checkpoint” OR “therapy.” Only articles directly addressing gastric cancer and microbiome-related mechanisms or interventions were included, while studies unrelated to gastric pathology were excluded.

### Risk factors and their relationship with the microbiome

Among the most influential determinants of gastric carcinogenesis are microbial and environmental risk factors that reshape the gastric ecosystem and disrupt mucosal homeostasis. The best-characterized of these is *H. pylori* infection, which contributes to tumorigenesis through chronic gastritis, sustained oxidative stress, and epithelial barrier disruption (11, 12). Beyond *H. pylori*, metagenomic studies of gastric cancer tissues have revealed increased abundance of genera such as *Fusobacterium*, *Streptococcus*, and *Lactobacillus*, which may exacerbate carcinogenesis by producing pro-inflammatory metabolites, altering intragastric pH, and activating oncogenic signaling cascades. Their presence in *H. pylori*-negative tumors further implicates microbial dysbiosis as an independent driver of malignant transformation (8, 13). Expanding this view, both gastric and intestinal microbiomes are now recognized as modulators of cancer susceptibility via regulation of immune signaling, epithelial proliferation, and production of genotoxic or immunomodulatory metabolites. In gastric cancer, specific microbial configurations correlate with activation of tumor-promoting pathways such as NF-κB and IL-6/STAT3, which enhance tumor growth, angiogenesis, and immune evasion. Moreover, microbiome composition has been shown to influence the efficacy of systemic therapies, including immunotherapy, by modulating inflammatory responses, immune activation thresholds, and drug metabolism (13–15). A rapidly emerging field of interest is the intratumoral microbiome, viable bacteria and microbial DNA detected within tumor tissues, that appears to actively participate in cancer biology by modulating local immune dynamics, sustaining oncogenic signaling, and impacting therapeutic response. Specific taxa have been linked to tumor aggressiveness, recurrence, and clinical prognosis, offering new avenues for biomarker discovery and microbiome-guided therapies (16). In parallel, lifestyle and environmental exposures, including tobacco use, alcohol intake, and diets rich in salt and ultra-processed foods, profoundly shape the gastric microbiota. These factors not only compromise epithelial integrity but also promote the expansion of pro-inflammatory and oncogenic microbial populations. Experimental models support that such exposures reduce microbial diversity and activate NF-κB and STAT3 signaling, thereby amplifying the pro-tumorigenic potential of host-environment-microbiome interactions (17–19).

### The role of the microbiota in gastric carcinogenesis

Chronic infection with *H. pylori* is universally recognized as the principal risk factor for gastric cancer, particularly non-cardia gastric adenocarcinoma. Beyond its direct oncogenic effects, *H. pylori* profoundly alters the gastric microbiota by reducing microbial diversity and fostering a sustained pro-inflammatory milieu conducive to neoplastic transformation (12). Dysbiosis, characterized by imbalances in microbial composition and function, has been mechanistically associated with the sequential progression from chronic gastritis to intestinal metaplasia, dysplasia, and ultimately invasive carcinoma (20). This dysregulated microecosystem, shaped by host-microbe-environment interactions, creates conditions favorable for tumor initiation and progression. Microbial composition is highly dynamic and influenced by multiple factors, including age, sex, diet, lifestyle, geographic location, *H. pylori* infection, mucosal inflammation, delivery mode at birth, and the use of antimicrobials or proton pump inhibitors (21, 22).

The interplay between *H. pylori* and the resident gastric microbiota further influences host immune responses and epithelial transformation, as *H. pylori* exhibits capabilities to evade immune surveillance, induce DNA damage, and disrupt mucosal equilibrium—all critical steps in malignant evolution (23, 24). Moreover, recent analyses of gastric and salivary microbiomes before and after gastrectomy have revealed coordinated microbial shifts, indicating a functional axis between oral and gastric microbial ecosystems in gastric cancer patients (25). Gastric microbial dysbiosis has also been implicated in modulating the efficacy of cancer therapies, as specific microbial signatures influence inflammatory signaling, immune activation, and drug metabolism, ultimately affecting responses to chemotherapy and immunotherapy (26–28).

From a public health perspective, eradication of *H. pylori* represents one of the most effective and evidence-based preventive measures against gastric cancer. The protective effect is strongest when eradication occurs early, before irreversible precancerous lesions such as atrophic gastritis or intestinal metaplasia have developed, since removal of the bacterium halts inflammation, restores mucosal homeostasis, and reduces the risk of progression to neoplasia (29, 30).

### Gastric microbiome and its impact on gastric cancer

#### Gastric microbiota composition and functions under normal conditions

Under normobiotic conditions, the gastric environment hosts a specialized microbial community adapted to withstand the extreme acidity of the stomach. Dominated by acid-tolerant genera such as *Streptococcus mitis*, *Lactobacillus reuteri*, *Rothia mucilaginosa*, and *Neisseria mucosa*, this ecosystem persists through biofilm formation and urease-mediated buffering mechanisms that support colonization within mucosal niches (31). These microbes maintain mucosal integrity and contribute to local immune homeostasis by engaging with epithelial cells and producing antimicrobial compounds (32). Population-level variability in gastric microbiota composition is influenced by factors such as altitude, ethnicity, genetics, diet, and early-life microbial exposure (33). The gut microbiota plays a pivotal role in the breakdown of nutrients. These microbial communities secrete enzymes capable of degrading complex carbohydrates that escape digestion in the upper gastrointestinal tract. Through fermentation of dietary fibers, they generate short-chain fatty acids (SCFAs), which not only provide energy to intestinal epithelial cells but also modulate lipid and glucose homeostasis and exert anti-inflammatory effects (28, 34). Functionally, this microbial consortium supports xenobiotic detoxification, vitamin biosynthesis, nitrogen metabolism, and redox balance, and inhibits colonization by pathogens like *Clostridium difficile* and *Enterobacteriaceae* (20, 35). Gastric epithelial pattern recognition receptors such as TLR2 and TLR4 mediate immune surveillance, driving mucin production, antimicrobial peptide release, and macrophage polarization to sustain microbial equilibrium (36, 37).

The early-life establishment of the gastric microbiome is vulnerable to disruption by antibiotics, PPIs, and low-fiber diets, which diminish taxa like *Lactobacillus* and *Faecalibacterium* and reduce levels of butyrate and propionate, key epigenetically active metabolites involved in gene regulation and immune modulation (38, 39). Recent multi-omics approaches have revealed that the healthy gastric microbiota is metabolically active, enriched in pathways for polyamine biosynthesis, sulfur metabolism, and heterocyclic amine detoxification, which may collectively reduce neoplastic risk (40, 41).

#### Factors influencing the gastric dysbiosis

Gastric dysbiosis, defined as alterations in the composition and function of the host-associated microbiota, has emerged as a critical factor in cancer development. Gastric dysbiosis arises from a complex interplay of dietary, microbial, pharmacological, and host-related factors that collectively reshape the gastric microenvironment and promote carcinogenesis. Diets rich in animal protein and saturated fats selectively enrich proteolytic and nitrosating bacteria, while fiber- and polyphenol-rich diets support beneficial taxa and SCFA production, contributing to mucosal integrity and anti-inflammatory signaling (42, 43). Broad-spectrum antibiotics markedly reduce microbial diversity and functional redundancy, facilitating opportunistic overgrowth by species such as *Clostridioides difficile* and *Enterococcus faecalis* (20, 44). Gastric acid acts as a primary defense, but its neutralization through prolonged PPI use raises gastric pH and permits retrograde colonization by intestinal microbes such as *Escherichia coli* and *Enterobacter cloacae*, which generate nitrites and other genotoxins (45, 46). In elderly individuals, hypochlorhydria, immune senescence, and comorbidities further exacerbate dysbiosis. Notably, co-exposures, such as low-fiber diets, PPI use, and CagA-positive *H. pylori* infection, exert synergistic effects, amplifying oxidative stress and activating microbial pathways associated with carcinogenesis (41, 47).

Dysbiotic states within the gastric microenvironment facilitate the accumulation of carcinogenic metabolites, most notably N-nitroso compounds (NOCs). These compounds may originate from exogenous dietary sources, such as processed meats, smoked foods, and nitrate-rich vegetables, or be generated endogenously via microbial nitrosation reactions, particularly under conditions of hypochlorhydria. In a reduced-acid gastric environment, enriched nitrate- and nitrite-reducing bacteria catalyze the formation of NOCs from dietary precursors, amplifying genotoxic stress and epithelial damage. These mechanisms have been consistently linked to increased gastric cancer risk and underscore the metabolic consequences of microbiota-driven ecological shifts in the stomach (47–49). Enriched taxa in gastric cancer, including *Veillonella*, *Clostridium*, *Haemophilus*, *Staphylococcus*, *Neisseria*, *Lactobacillus*, and *Nitrospirae*, exhibit elevated nitrate/nitrite reductase activity, further enhancing NOC production and epithelial DNA damage (47, 50). Additionally, lactic acid bacteria, frequently abundant in gastric tumors, contribute to carcinogenesis by increasing ROS and lactate levels, promoting immune tolerance, and facilitating epithelial–mesenchymal transition (49, 51).

#### Alterations of the microbiota in gastric cancer and dysbiosis

Emerging evidence shows that intratumoral and intracellular bacteria actively modulate epithelial–mesenchymal transition, angiogenesis, stromal remodeling, immune evasion, and therapy response. Spatial multi-omic approaches reveal microbial hubs linked to immunosuppression and pre-metastatic niche formation, reframing tumor microbial biomass as a targetable driver of metastasis rather than a passive bystander (52).

In parallel, the transition from gastric eubiosis to dysbiosis is characterized by marked ecological shifts, including reduced alpha diversity and selective enrichment of pro-inflammatory species across the metaplasia–dysplasia–carcinoma sequence (53, 54). This dysbiotic state is accompanied by enhanced microbial metabolic pathways involved in nitrogen compound transformation, LPS biosynthesis, and heterocyclic amine formation, driving sustained inflammation via NF-κB, IL-1β, and TNF-*α* signaling (24, 55). At the network level, dysbiosis is characterized by a depletion of beneficial SCFAs-producing genera, including *Lactobacillus*, *Bifidobacterium*, and *Faecalibacterium*, alongside an enrichment of pro-tumorigenic taxa such as *Fusobacterium*, *Veillonella*, and *Peptostreptococcus*. These alterations have been linked to the activation of matrix metalloproteinases (MMPs) and the hypermethylation of tumor (47, 56). In mouse models, *H. pylori*-induced dysbiosis triggers systemic immune alterations along the gut–stomach axis, impairing antitumor immunity (57). Post-gastrectomy microbiota studies corroborate these findings, revealing persistent dysbiosis, loss of beneficial microbes like *Akkermansia muciniphila*, and emergence of ROS-producing pathobionts such as *E. coli* and *E. faecalis*, which impair mucosal regeneration (Table 1) (25, 58).

**Table 1:** Key microbial drivers and mechanisms in gastric cancer.

Microorganism	Localization/context	Associated taxon	Main mechanism/impact	Therapeutic link	Evidence type	Oncologic consequence
*Helicobacter pylori* (urease, CagA, VacA)	Gastric mucosa; non-cardia adenocarcinoma	*H. pylori*	Chronic inflammation, ROS, immune evasion; CagA and VacA disrupt cell polarity and signaling	Eradication reduces incidence and metachronous cancer; target for prevention	Preclinical + Clinical (RCTs/observational)	Major etiologic factor; worse prognosis if persistent
*Fusobacterium nucleatum* (FadA/Fap2)	Gastric tumor tissue	*F. nucleatum*	E-cadherin binding - NF-κB/IL-6 activation; Fap2 mediates immune evasion	Potential modulation target	Translational/Clinical (association studies)	Poor survival; increased aggressiveness
*Streptococcus anginosus* (TMPC–ANXA2)	Gastric epithelium; AGS/MKN1 cell lines	*S. anginosus*	Adhesion/invasion; MAPK activation; enhanced proliferation	-	*In vitro* + Observational	Pro-proliferative microenvironment
*Lactobacillus fermentum* (strain-dependent)	Stomach; murine models	*L. fermentum* (e.g., UCO-979C)	Anti-inflammatory or pro-tumorigenic effects depending on strain; lactate/ROS modulation	Candidate probiotic (context-dependent)	Preclinical	Dual role depending on strain and host context
*Propionibacterium acnes*	*H. pylori*–negative gastric cancer	*P. acnes* (Cutibacterium)	M2 macrophage polarization via TLR4/PI3K/Akt; IL-10/CCR-2 axis	-	Translational	Immunosuppression; tumor progression

CagA, cytotoxin-associated gene A; VacA, vacuolating cytotoxin A; RCT, randomized controlled trial; FadA, *Fusobacterium* adhesin A; Fap2, *Fusobacterium* autotransporter protein 2; NF-κB, nuclear factor kappa-light-chain-enhancer of activated B cells; IL-6, interleukin-6; TMPC–ANXA2, tumor-promoting complex between *Streptococcus anginosus* and annexin A2; AGS/MKN1, human gastric adenocarcinoma cell lines; MAPK, mitogen-activated protein kinase; UCO-979C, *Lactobacillus fermentum* strain UCO-979C; M2, alternatively activated macrophage; TLR4, Toll-like receptor 4; PI3K, phosphoinositide 3-kinase; Akt, protein kinase B; IL-10, interleukin-10; CCR-2, C-C chemokine receptor type 2; *H. pylori, Helicobacter pylori*; *F. nucleatum, Fusobacterium nucleatum; S. anginosus, Streptococcus anginosus; L. fermentum, Lactobacillus fermentum; P. acnes, Propionibacterium acnes* (currently *Cutibacterium acnes*); GC, gastric cancer.

#### Interactions between the gastric and intestinal microbiome in tumor progression

The gastrointestinal microbiome constitutes a dense and metabolically active ecosystem that extends from the oral cavity to the colon, shaping host physiology through fermentation, metabolite production, immune modulation, and epigenetic signaling (59–61). Within this system, the gut–stomach microbial axis plays a critical role in gastric tumorigenesis. Disruption of microbial homeostasis, induced by antibiotics, proton pump inhibitors (PPIs), dietary changes, or infection, leads to dysbiosis, marked by loss of commensal taxa, reduced microbial diversity, and expansion of pathogenic organisms. This dysbiosis fosters systemic immune reprogramming, wherein gut-derived regulatory T cells and dendritic cells migrate to the gastric mucosa, promoting immune evasion and chronic inflammation (43, 47, 62). Moreover, SCFAs such as butyrate and acetate, produced by beneficial intestinal bacteria, regulate epithelial proliferation and apoptosis in the stomach; their depletion contributes to a pro-inflammatory and tumor-permissive microenvironment (43, 63). Increased intestinal permeability (“leaky gut”) allows translocation of microbial components like lipopolysaccharide (LPS), activating TLR4/NF-κB signaling in gastric tissues and amplifying oncogenic inflammation (64, 65). Hypochlorhydria, whether due to chronic PPI use or gastric atrophy, further facilitates retrograde colonization by intestinal bacteria such as *E. faecalis* and *B. fragilis*, which induce oxidative stress, DNA damage, and epithelial dysplasia (66, 67).

A circadian layer modulates host-microbe-immune interactions in gastric oncogenesis. Peripheral clocks, synchronized by zeitgebers such as feeding and light, regulate barrier function, metabolism, and inflammation; their misalignment fosters dysbiosis and impaired antitumor immunity. These findings highlight chronotherapy and time-aware approaches as promising strategies to optimize microbiome-driven responses in gastric cancer (68). In parallel, multi-omics studies integrating metagenomics, transcriptomics, and metabolomics have revealed that microbial structure and function are shaped by local gastric and intestinal physicochemical conditions, particularly pH and moisture, which alter bacterial gene expression, metabolite output, and virulence (69, 70). Notably, a progressive loss in gut microbial diversity correlates with tumor advancement, highlighting its potential as a prognostic biomarker (71).

Although *H. pylori* remains the principal agent in gastric carcinogenesis, emerging evidence implicates gut microbiota composition, particularly the presence of *Akkermansia muciniphila* and *Ruminococcus* spp., as a determinant of response to immune checkpoint inhibitors, underscoring the importance of microbiome-driven immunomodulation in therapeutic outcomes (47, 72). Additionally, microbiota-derived metabolites such as SCFAs, bile acids, and polyamines can influence host oncogenic pathways, epigenetic regulation, and immune tone, reinforcing the concept of a dynamic gut-stomach microbial axis in gastric cancer progression (73, 74). These insights underscore the necessity of personalized microbiome profiling to identify predictive microbial signatures and develop targeted interventions in stomach oncology.

### Bacteria linked to carcinogenesis

#### The oncogenic role of *Helicobacter pylori* in gastric carcinogenesis

Tumor development in the stomach emerges from cumulative carcinogenic exposures and host–microbe interactions, with *H. pylori* representing the most established microbial risk factor for gastric adenocarcinoma. Although it colonizes more than 60% of adults, fewer than 2% develop gastric cancer, underscoring strain-specific virulence and host context. As a Gram-negative, microaerophilic organism, *H. pylori* relies on urease to buffer gastric acidity and persist within mucosal niches; beyond pH neutralization, urease activates neutrophils and sustains pro-inflammatory signaling, thereby contributing to epithelial injury and carcinogenic priming (12, 75, 76).

Immune evasion and chronic inflammation. *H. pylori* modifies LPS and flagellin to dampen TLR4/TLR5 recognition, blunting innate activation (77, 78). OipA impairs dendritic cell maturation and antigen presentation, curtailing effective T-cell priming; high OipA expression in *H. pylori* is positive GC further implicates immunoevasion (79). This dual program, sustained IL-8–driven inflammation with concurrent immune suppression, creates a niche permissive to DNA damage, epithelial remodeling, and stepwise malignant evolution (80, 81).

Among *H. pylori* virulence factors, cytotoxins CagA and VacA play central roles in oncogenic reprogramming. CagA, encoded within the cag pathogenicity island and delivered into gastric epithelial cells via a type IV secretion system, undergoes phosphorylation at tyrosine residues by host kinases. Activated CagA interacts with SHP2 and other signaling molecules, deregulating cell proliferation, migration, and polarity. Beyond classical signaling, CagA induces lipidomic rewiring by upregulating AGPS and AGPAT3, enhancing synthesis of polyunsaturated ether phospholipids (PUFA-ePLs). These metabolites sensitize non-malignant cells to ferroptosis but, paradoxically, in tumor cells promote accumulation of palmitoyl-CoA, which stabilizes PD-L1 through S-palmitoylation, thereby supporting immune escape. VacA further compromises mitochondrial integrity, impairs epithelial junctions, and dampens T-cell activation, collectively potentiating chronic injury. Together, CagA and VacA amplify oncogenic networks including NF-κB and IL-6/STAT3, fueling angiogenesis, proliferation, and immune suppression (49, 82).

Persistent *H. pylori* infection is tightly linked to the Correa cascade, which describes progression from chronic gastritis through atrophy, intestinal metaplasia, dysplasia, and eventually carcinoma. Central to this process is epigenetic remodeling of gastric stem and progenitor cells, with widespread CpG island methylation silencing tumor suppressor genes and altering differentiation programs (50, 83). In addition, *H. pylori* perturbs autophagic flux, enabling survival of genetically altered cells that would otherwise be eliminated, thereby facilitating progression toward invasion and metastasis (73, 84). Importantly, even after eradication, dysbiotic alterations in the gastric microbiota often persist, suggesting that *H. pylori* not only acts as an initiator but also as a community-level architect of microbial ecosystems that sustain carcinogenic risk (24).

### Other bacteria associated in gastric carcinogenesis

*Fusobacterium nucleatum: F. nucleatum* is a Gram-negative anaerobic bacterium primarily residing in the oral cavity, where it is linked to periodontal disease. However, its detection in gastrointestinal and extraintestinal tumors, including those of the colon, esophagus, pancreas, and stomach, has positioned it as a potential microbial contributor to carcinogenesis. In gastric cancer, high abundance of *F. nucleatum* in tumor tissue correlates with poor prognosis and reduced overall survival (85, 86). Mechanistically, the bacterium promotes immune evasion by inhibiting T-cell activity and facilitates tumor progression through proinflammatory signaling. Its surface adhesin FadA binds to E-cadherin on epithelial and endothelial cells, triggering NF-κB and IL-6–mediated inflammatory cascades. Additionally, *F. nucleatum* expresses Fap2, a galactose-binding lectin-like protein that recognizes tumor-associated glycans, enabling selective localization to tumor sites and further promoting immune suppression (71, 87).

*Streptococcus anginosus: S. anginosus* is a member of the oral microbiota but has been increasingly implicated in gastric pathologies. Epidemiological data suggest a link between poor oral hygiene and elevated gastric cancer risk, reinforcing the hypothesis that the oral-gastric microbial axis plays a critical role in carcinogenesis (88). *S. anginosus* is abundant across mucosal niches, tongue, gingiva, hard palate, and can colonize the gastric epithelium, where it recruits neutrophils and monocytes, contributing to an inflammatory microenvironment conducive to neoplastic transformation. *In vitro* co-culture experiments using AGS and MKN1 gastric cancer cell lines demonstrated that *S. anginosus* adheres to and infiltrates cancer cells. This interaction is mediated by the TMPC protein, which binds to the annexin A2 (ANXA2) receptor on host cells, activating the MAPK signaling pathway and promoting proliferation (47, 89, 90).

*Lactobacillus fermentum:* while classically viewed as a commensal with probiotic potential, certain *Lactobacillus* species, including *Lactobacillus fermentum*, exhibit dynamic roles in gastric cancer depending on strain-specific functions and disease context. Co-occurrence analyses reveal that *L. fermentum* and *L. sali*var*ius* are present across various stages of gastric carcinogenesis, from early-stage disease to precancerous lesions and invasive tumors, highlighting their potential immunomodulatory roles (89). Importantly, evidence of *Lactobacillus* enrichment within GC tissues and lactate-driven metabolic and immunologic reprogramming suggests that not all lineages act uniformly as “protective”; rather, tumor-associated niches may favor *Lactobacillus* overgrowth with pro-tumor correlates in specific contexts (91). Interestingly, specific strains such as *L. fermentum* UCO-979C, isolated from the human stomach, demonstrate anti-*H. pylori* and anti-inflammatory activities, attenuating chronic *H. pylori*-induced inflammation by modulating epithelial immune responses; in murine and cell-based models, UCO-979C reduces pro-inflammatory cytokines/chemokines and interferes with urease activity and adherence, changes associated with improved mucosal defense (92, 93). Thus, the seemingly contradictory reports are reconciled by strain-level heterogeneity (e.g., UCO-979C vs. non-characterized *Lactobacillus* overgrowth) and by the anatomical/microenvironmental niche (healthy vs. tumor tissue) (94).

### Molecular mechanisms of microbiota in gastric carcinogenesis

#### Chronic inflammation and epithelial damage

*H. pylori* infection induces persistent inflammation of the gastric mucosa, triggering a cascade of molecular alterations in gastric epithelial cells that culminate in glandular atrophy, intestinal metaplasia, dysplasia, and ultimately gastric adenocarcinoma. Central to this process is the activation of pro-inflammatory signaling pathways such as NF-κB and IL-6/STAT3, as well as the generation of reactive oxygen species (ROS), all of which contribute to chronic mucosal injury, epithelial dedifferentiation, and DNA damage (12, 49).

#### *Helicobacter pylori* virulence factors and their oncogenic mechanisms

Among the virulence factors of *H. pylori*, the cytotoxins CagA and VacA play pivotal roles in gastric carcinogenesis by manipulating host cellular pathways, disrupting immune surveillance, and inducing chronic inflammation. CagA, encoded within the *cag* pathogenicity island (cagPAI), is delivered into gastric epithelial cells via a type IV secretion system. Once internalized, its C-terminal region, rich in tyrosine phosphorylation motifs, is phosphorylated by host kinases, enabling interaction with SHP2, a phosphatase involved in cell proliferation and migration. This aberrant signaling promotes epithelial transformation and oncogenic reprogramming (95, 96). Moreover, CagA disrupts host lipid metabolism by upregulating AGPS and AGPAT3, thereby enhancing the synthesis of polyunsaturated ether phospholipids (PUFA-ePLs) that sensitize non-malignant cells to ferroptosis. In tumor cells, CagA-driven lipogenic remodeling increases palmitoyl-CoA availability, which promotes the S-palmitoylation and stabilization of PD-L1 at the plasma membrane. Stabilized PD-L1 then binds PD-1 on cytotoxic T lymphocytes, suppressing their activity and enabling immune escape. CagA also upregulates squalene epoxidase (SQLE), further reinforcing sterol biosynthesis and oncogenic lipid remodeling (97–99).

Mechanistically, there is direct evidence that VacA can modulate E2F1 in immune cells: in human dendritic cells, VacA restored E2F1 expression suppressed by LPS, restraining maturation and sustaining an immature phenotype (100). By contrast, in gastric epithelial models, no study has demonstrated VacA-driven upregulation of E2F1. Instead, VacA induces apoptosis and G1 arrest through p53/p21/Bax signaling and triggers ER-stress–dependent autophagic cell death in AGS cells (101, 102). More broadly, *H. pylori* exposure impedes the G1–S cell cycle transition by downregulating p27^Kip1 and altering c-fos SRE activity, perturbations that converge on, yet do not directly demonstrate, dysregulation of the RB–E2F axis. Accordingly, we frame “from VacA to E2F1 upregulation in gastric epithelium” as a plausible, yet unproven, epithelial mechanism supported indirectly by VacA’s epithelial effects and by the centrality of RB–E2F circuitry in gastric tumorigenesis (102–104). In addition, VacA has been linked to epigenetic remodeling, including miR-210 silencing, which may converge on proliferative pathways relevant to oncogenesis (105, 106).

Innovative therapeutic strategies, such as sonodynamic therapy with biodegradable Ver-PLGA@Lecithin nanoparticles, have recently shown promise in neutralizing VacA without perturbing the intestinal microbiota. In *H. pylori*-infected mice, this approach not only inactivated VacA but also promoted expansion of protective *Lactobacillus* species (12, 107).

Complementing these bacterial mechanisms, Epstein–Barr virus (EBV)–positive gastric cancer highlights how oncogenic viruses reprogram the host epigenome, altering DNA methylation, histone modifications, chromatin accessibility, and non-coding RNAs to enforce immune evasion and stemness-like programs. This viral epigenetic imprinting provides mechanistic support for combined epigenetic-immunotherapeutic approaches in GC subsets with viral etiologies, including the rational integration of DNMT/HDAC inhibitors and oncolytic virotherapy to enhance antigenicity and checkpoint sensitivity (108).

#### Non-*Helicobacter pylori* microbial contributors

Beyond *H. pylori*, a growing number of studies implicate diverse non-*H. pylori* microbes in shaping the gastric tumor microenvironment through both pro-inflammatory and immunosuppressive mechanisms. *Propionibacterium acnes*, frequently enriched in GC tissues, especially in *H. pylori*–negative cases, promotes M2 macrophage polarization via TLR4/PI3K/Akt signaling, resulting in IL-10 and CCR-2 secretion that fosters immunosuppression and tumor progression. In lymphocytic gastritis associated with *P. acnes*, elevated IL-15 levels suggest a pro-inflammatory trigger for gastric malignancy. Similarly, colonization by *Lactobacillus murinus*, *Clostridium*, and *Streptococcus salivarius* in INS-GAS mice upregulated inflammation- and oncogenesis-related gene expression, accelerating intraepithelial neoplasia (109, 110). Additional taxa enriched in GC tissues have been correlated with activation of immune pathways and tumor growth in translational studies (111, 112). Collectively, these findings underscore that multiple microbial species act beyond *H. pylori* to modulate gastric inflammation, immune polarization, and oncogenic signaling, highlighting the need to include non-*H. pylori* taxa in comprehensive models of gastric carcinogenesis.

#### Modulation of microbiome-immune interaction

The intricate crosstalk between the gastric microbiota and the host immune system plays a pivotal role in modulating gastric carcinogenesis. Beyond *H. pylori*, emerging data highlight a broader microbial ecosystem influencing immune dynamics in the gastric mucosa, contributing to either immune surveillance or tumor-promoting inflammation. *H. pylori* initiates chronic gastritis by inducing both innate and adaptive responses, but over time promotes immune evasion through mechanisms such as suppression of Th1 cytokines and enhancement of Th2-skewed immunity via group 2 innate lymphoid cells (ILC2s) (113, 114). ILC2s, highly enriched in the gastric mucosa, are uniquely dependent on local microbiota and are rapidly expanded in response to *H. pylori* through IL-7 and IL-33 signaling axes, contributing to IgA production but also sustaining a Th2-biased, immunosuppressive microenvironment. This immunosuppressive polarization is further reinforced by the expansion of myeloid-derived suppressor cells and M2 macrophages, both of which are supported by ILC2 cytokines such as IL-5 and IL-13 (115, 116). The IL-33/IL-13 cascade, particularly, has been implicated in the induction of spasmolytic polypeptide-expressing metaplasia, a precursor lesion in gastric cancer, through recruitment and polarization of alternatively activated macrophages (117). Moreover, *Propionibacterium acnes*, enriched in *H. pylori*-negative tumors, can induce M2 macrophage polarization via TLR4/PI3K/Akt signaling, further contributing to immune suppression and tumor progression (114). Neutrophils, traditionally seen as antimicrobial effectors, have gained recognition for their plasticity in cancer, displaying both pro- and anti-tumor roles depending on cues from the tumor microenvironment—many of which are microbiota-derived (116). Collectively, the modulation of microbiome-immune interactions involves a multifaceted network of innate lymphoid cells, regulatory cytokines, tumor-associated macrophages, and microbial metabolites, shaping an immunological niche that determines the trajectory from chronic inflammation to neoplastic transformation (50, 118). Understanding these dynamic immunological shifts opens new avenues for microbiota-targeted immunotherapies and early interventions in gastric cancer.

### Therapeutic strategies based on microbiota modulation

#### Use of probiotics and prebiotics

The therapeutic potential of probiotics and prebiotics in the context of GC has gained increasing attention due to their ability to modulate host immunity, restore microbial homeostasis, and influence tumor biology. Probiotic strains, particularly those from the *Lactobacillus* and *Bifidobacterium* genera, have demonstrated efficacy in reducing pro-inflammatory responses, promoting dendritic cell maturation, enhancing cytokine-mediated immune activation, and increasing tumor cell apoptosis through modulation of key signaling pathways such as NF-κB and PI3K/Akt/mTOR (111, 119, 120). *Lactobacillus* strains have shown inhibitory effects on GC development by attenuating inflammatory cascades; however, their heightened abundance in advanced-stage gastric tumors raises concerns about context-dependent roles and underscores the need for strain-specific evaluation (121).

Conversely, *Bifidobacterium* effects are strain and indication-specific. Selected strains (e.g., *B. longum*, *B. breve*) have been linked to enhanced antigen presentation and CD8^+^ T-cell priming, associations that align with improved ICI activity in melanoma and other solid tumors, whereas other strains (e.g., *B. infantis*, *B. bifidum*) can expand Foxp3^+^ Tregs and favor immune tolerance under particular inflammatory conditions (oncology context dependence). This clarification reconciles previously described “immunity-enhancing” versus “immune-suppressive” effects and supports a precision, strain-resolved approach to probiotic use in GC (122, 123).

Beyond immunomodulation, probiotics have demonstrated protective effects against gastrointestinal toxicity induced by anticancer therapies. For instance, *Lactobacillus acidophilus* and *Bifidobacterium longum* can alleviate radiation-induced diarrhea, and certain probiotic formulations reduce oxaliplatin-associated intestinal damage in both murine models and clinical settings (7, 124). Prebiotics, by promoting the selective growth of beneficial gut microbes, enhance the production of SCFAs, improve micronutrient absorption, regulate immune function, and support metabolic homeostasis, thus improving therapeutic efficacy and resilience during cancer treatment (125, 126). Nevertheless, due to the complex and sometimes paradoxical behavior of specific probiotic strains, some of which are enriched within gastric tumor tissues, caution is warranted when implementing probiotic-based interventions in GC. Individualized and mechanistically informed strategies are essential to avoid exacerbating tumor-promoting pathways while maximizing therapeutic benefit (127).

#### Microbiota and chemotherapy

Growing evidence supports that the gut microbiota plays a critical role in shaping the efficacy and toxicity of chemotherapeutic agents. Microbial metabolites and immune modulation mediate these interactions, where dysbiosis can impair drug response and foster resistance (128). For instance, *Lactobacillus* species and their secreted metabolites enhance the antitumor activity of 5-fluorouracil (5-FU) and can reverse resistance through immunomodulation and regulation of intracellular signaling pathways (129). Butyrate, a short-chain fatty acid derived from microbial fermentation, promotes the efficacy of oxaliplatin by enhancing CD8^+^ T cell cytotoxicity, while the efficacy of both oxaliplatin and cisplatin is significantly impaired in antibiotic-treated mice, indicating the microbiota’s indispensable role in supporting host immune responses during chemotherapy. Importantly, disruption of the gut ecosystem with broad-spectrum antibiotics has been associated with poor response and higher toxicity in patients undergoing chemotherapy, further reinforcing the rationale for maintaining or restoring microbiome equilibrium throughout oncologic treatment (130, 131).

Fecal microbiota transplantation (FMT) has emerged as a direct and robust approach to reshape the gut microbiota and potentiate chemotherapy outcomes. Clinical data suggest that FMT from healthy donors, particularly those with metabolically enriched microbiomes, may enhance response to capecitabine and oxaliplatin in patients with metastatic esophagogastric cancer, improving progression-free survival and immune function (132, 133). Additionally, data from preclinical models and early human trials highlight the potential of FMT to alleviate chemotherapy-induced intestinal toxicity, restore microbial balance, and support mucosal healing, representing a dual role in both efficacy enhancement and toxicity mitigation (127).

#### Microbiota and immunotherapy

The gut microbiota plays a central role in shaping host responses to immune checkpoint inhibitors (ICIs), influencing antitumor immunity through mechanisms such as dendritic cell maturation, antigen presentation, and T cell priming (129, 134).

The microbiome–ICI association is clade- and context-specific rather than “*Clostridium*-wide.” In melanoma and mixed solid tumors, butyrate-producing Clostridia, mainly Ruminococcaceae/Lachnospiraceae such as *Faecalibacterium prausnitzii*, *Eubacterium rectale*, and *Roseburia* spp., have been associated with anti–PD-1/PD-L1 response and improved T-cell priming; however, these findings are not universal across *Clostridium sensu lato* and remain unvalidated in GC (135, 136). Conversely, other Clostridia clusters IV/XIVa can expand Foxp3^+^ Tregs and may attenuate antitumor immunity, underscoring strain- and disease-specific effects. GC-specific data remain limited; emerging studies in gastrointestinal cohorts identify taxa such as *Akkermansia muciniphila* and *Dorea formicigenerans* as candidate biomarkers of ICI benefit, rather than a uniform *Clostridium* signal. Accordingly, we refer to “selected butyrate-producing Clostridia (e.g., Ruminococcaceae/Lachnospiraceae)” and specify cancer type and ICI class when describing associations, avoiding universal claims in GC (136–138).

The role of *Bifidobacterium* in this context is complex, strain-specific, and tumor-dependent, which accounts for the apparent discrepancies across studies. For instance, *B. breve* and *B. longum* have been associated with enhanced antitumor immunity by promoting dendritic cell–derived IL-12 signaling and cross-reactive CD8^+^ T-cell priming (139, 140). In clinical and preclinical models, *B. breve* correlates with improved progression-free survival in patients receiving anti–PD-1 plus chemotherapy (136–138), while *B. longum* has been repeatedly enriched in responders to PD-1 blockade, enhancing dendritic cell function and intratumoral CD8^+^ T-cell accumulation (122, 141). These findings align with seminal studies demonstrating microbiome-driven modulation of immune checkpoint efficacy in melanoma (142), clarifying why “beneficial” and “suppressive” effects can both be accurate depending on strain, disease state, and therapy.

Conversely, some species/strains such as *B. infantis* and *B. bifidum* have been linked to expansion of Foxp3^+^ regulatory T cells via IL-10 and TGF-*β* signaling, which may foster immune tolerance and attenuate ICI efficacy, particularly in chronic inflammatory states or tumor-specific contexts such as gastric cancer, where available data remain limited (142–144). Beyond compositional differences, dysbiosis itself—whether triggered by antibiotics, inflammation, or diet, can impair antigen-specific T cell responses by disrupting cytokine networks, compromising immune surveillance, and reducing therapeutic effectiveness (129, 145).

Given these dynamics, interventions aimed at restoring favorable microbial profiles are under investigation. Fecal microbiota transplantation (FMT) has demonstrated the capacity to convert non-responders into responders in melanoma, mitigate ICI-related colitis, and is now being explored in gastrointestinal and prostate cancers (132, 146). Collectively, these findings underscore that while selected *Bifidobacterium* strains such as *B. breve* and *B. longum* may potentiate CD8^+^ T cell–mediated immunity, others promote regulatory pathways, emphasizing the need to account for microbial heterogeneity, tumor context, and limited gastric-specific evidence when interpreting their impact on immunotherapy outcomes (123, 147, 148).

#### Diet and gastric microbiome

Dietary composition is one of the most influential and modifiable external factors shaping the human gastrointestinal microbiota. Long-term dietary patterns profoundly affect both the structure and function of the gastric and intestinal microbial communities, determining whether these interactions promote health or disease (7, 149). Adherence to a Western-style diet, characterized by high intake of saturated fats, refined sugars, and low dietary fiber, has been consistently associated with microbial dysbiosis and an increased risk of GC. This diet promotes the expansion of pro-inflammatory and potentially oncogenic taxa, disrupts mucosal immune equilibrium, and impairs microbial metabolite diversity (127). Additionally, high-fat diets increase gastric leptin expression, promoting the development of intestinal metaplasia, a known precancerous lesion. Cooking practices commonly associated with the Western diet, such as high-temperature grilling (150–300 °C) and nitrite-curing of meats, lead to the formation of mutagenic compounds like heterocyclic amines, which contribute to DNA damage and are implicated in gastric and colorectal carcinogenesis (150).

Conversely, high-fiber diets, prebiotic-rich foods, and consumption of fermented products that provide probiotic strains have shown protective effects by fostering the growth of SCFA-producing and anti-inflammatory bacteria, restoring microbial diversity, and reinforcing epithelial integrity—thus reducing GC risk (127, 151). Beyond dietary content, interventional strategies such as fasting and caloric restriction have shown promise in preclinical and clinical studies, where they modulate systemic metabolism, enhance antitumor immunity, and suppress tumor growth by reshaping the microbiome and its metabolomic output (111, 152). These interventions have also demonstrated the capacity to reduce chronic inflammation, improve immune surveillance, and mitigate chemotherapy-induced toxicity, offering a valuable adjunct to conventional anticancer treatments (153, 154).

#### Eradication of *Helicobacter pylori*

Given that *H. pylori* is the primary risk factor for the development of GC, its early detection and eradication constitute the cornerstone of both preventive and therapeutic strategies (155, 156). Eradication therapy not only mitigates the inflammatory and oncogenic stimuli driven by *H. pylori*, but also contributes to the partial restoration of the gastric microbial ecosystem. Post-eradication studies have shown increased abundance of beneficial bacteria such as *Lactobacillus* and *Bifidobacterium*, as well as recovery of commensal phyla including *Firmicutes, Bacteroidetes*, *Actinobacteria*, and *Cyanobacteria* within the gastric mucosa (13, 157, 158). However, it is important to note that *H. pylori* eradication does not guarantee complete microbiota normalization or the elimination of carcinogenic risk. In some cases, microbial imbalance persists post-treatment, such as excessive enrichment of *Actinobacteria*, which may contribute to persistent dysbiosis and impaired mucosal recovery.

Randomized clinical trials have demonstrated that eradication is particularly effective in reducing GC incidence when administered before the development of precancerous lesions. Moreover, eradication therapy has been shown to decrease the risk of metachronous gastric cancer in patients who have undergone curative endoscopic resection for early-stage disease (29, 159). Despite these benefits, antibiotic resistance has emerged as a critical global challenge in *H. pylori* management. Resistance to commonly used antibiotics, including clarithromycin, metronidazole, and levofloxacin, is increasing, yet recent data on regional resistance trends remain scarce, hampering the development of effective, evidence-based eradication protocols (29, 160). Addressing this issue requires global surveillance systems, personalized susceptibility-guided therapies, and the exploration of adjunctive microbiota-preserving or restoring strategies to enhance long-term gastric mucosal health following eradication.

## Conclusions and future perspectives

The intricate interplay between the gastric and intestinal microbiota and gastric cancer (GC) pathogenesis has redefined our understanding of microbial contributions to oncogenesis beyond *Helicobacter pylori*. While *H. pylori* remains the most established microbial carcinogen, it cannot account for the full spectrum of gastric tumorigenesis. Multi-omics and mechanistic studies now support a paradigm in which dysbiosis of the gastric ecosystem, both local and systemic, drives tumor initiation, progression, and therapeutic outcomes (50, 128).

GC-associated dysbiosis is characterized by reduced microbial diversity, enrichment of pro-inflammatory and nitrosating bacteria, and loss of protective commensals. These shifts disrupt epithelial and immune homeostasis, promoting chronic inflammation, barrier dysfunction, and accumulation of genotoxic metabolites such as reactive oxygen species and N-nitroso compounds (8, 63). Activation of signaling pathways including NF-κB, IL-6/STAT3, and TLR cascades further sustains epithelial transformation, immune evasion, and malignant progression (57, 129).

Importantly, dysbiosis is not restricted to the stomach. Alterations along the gut–stomach axis extend systemic effects through translocation of metabolites, immune cells, and bacteria, reshaping gastric immunity and epithelial physiology (129, 161). Increased intestinal permeability enables microbial products such as LPS to trigger TLR4-driven inflammation, while depletion of SCFA-producing taxa like *Faecalibacterium* and *Roseburia* reduces mucosal repair and anti-inflammatory capacity, thereby amplifying carcinogenic risk (129).

The emerging recognition of intratumoral microbiota adds another layer to tumor–microbe interactions. Bacteria residing within gastric tumors can modulate therapeutic responses, reprogram immune activity, and contribute directly to DNA damage (162). Taxa such as *Fusobacterium nucleatum* and *Streptococcus anginosus* foster immune suppression, T-cell exhaustion, and pro-tumorigenic cytokine release, highlighting their potential as biomarkers and therapeutic targets (63, 76). Their spatial localization within tumors suggests potential for microbial biomarkers and targets for intervention.

From a clinical standpoint, the microbiome is now recognized as a modulator of response to chemotherapy, immunotherapy, and other systemic treatments (163). Antibiotic-induced dysbiosis has been associated with reduced efficacy of immune checkpoint inhibitors, highlighting the necessity of preserving microbial diversity during oncologic care (7). Conversely, beneficial microbes, particularly *Lactobacillus*, certain strains of *Bifidobacterium* have shown positive associations with ICI efficacy, and *Akkermansia muciniphila*, have been implicated in enhancing antitumor immunity via dendritic cell activation, improved antigen presentation, and increased infiltration of cytotoxic CD8^+^ T cells (50, 129). Likewise, instead of a generalized “Clostridium pro-ICI” signal, selected butyrate-producing Clostridia (e.g., *C. butyricum*) have been associated with improved responses in defined contexts; however, evidence in GC remains limited and heterogeneous (136).

Therapeutically, modulation of the microbiota offers a promising strategy to improve outcomes and mitigate treatment-related toxicity. Probiotic and prebiotic formulations have shown efficacy in restoring mucosal integrity, reducing inflammation, and enhancing chemotherapy efficacy, particularly with 5-fluorouracil and oxaliplatin. FMT is emerging as a powerful tool not only to restore microbial diversity but also to transfer responder phenotypes to immunotherapy non-responders. In gastrointestinal tumors, including GC, early-phase clinical trials suggest that FMT may enhance therapeutic response, modulate immune tone, and reduce adverse events associated with immune checkpoint blockade (7, 76).

Dietary interventions represent an accessible and non-invasive approach to shape microbial communities in a protective direction. High-fiber, polyphenol-rich diets have been shown to enrich SCFA-producing taxa, reduce systemic inflammation, and downregulate tumorigenic signaling pathways (63). In contrast, Western-style diets rich in saturated fats, processed meats, and low in fiber promote dysbiosis and the expansion of pro-oncogenic bacteria capable of nitrosation and ROS production (8, 76). Furthermore, fasting and caloric restriction have demonstrated microbiome-dependent benefits in tumor growth control, through modulation of microbial metabolite output and systemic immune reprogramming (7).

Despite these advances, challenges remain. The heterogeneity of microbial signatures across individuals, influenced by geography, diet, genetics, and comorbidities, complicates the development of universal therapeutic strategies. Additionally, the strain-specific behavior of certain taxa, such as *Lactobacillus fermentum*, which may act as both protective and tumor-promoting depending on context, underscores the need for precise microbial characterization and functional validation (50, 63).

Future research must prioritize longitudinal, multi-omics, and spatial analyses to differentiate causative microbial drivers from bystanders and to unravel host-microbe-metabolite interactions. Integrated approaches combining metagenomics, metabolomics, transcriptomics, and single-cell immune profiling will be instrumental in building predictive models and identifying microbial signatures of progression, response, and prognosis (50, 164). Equally, clinical trials incorporating microbiome endpoints alongside immune and metabolic markers are essential to validate microbiome-informed interventions in GC.

In conclusion, the microbiome is not a passive bystander but an active architect of the gastric tumor microenvironment and therapeutic response. From risk prediction and prevention to therapy sensitization and immune modulation, the microbiota represents a transformative axis in gastric cancer biology and clinical management. The integration of microbial diagnostics and interventions into precision oncology is within reach, provided that rigorous science, clinical translation, and patient-centered innovation converge.
